# The principles of pedestrian route choice

**DOI:** 10.1098/rsif.2022.0061

**Published:** 2022-04-06

**Authors:** Yunhe Tong, Nikolai W. F. Bode

**Affiliations:** Department of Engineering Mathematics, University of Bristol, Bristol BS8 1TW, UK

**Keywords:** route choice, pedestrian behaviour, decision-making, interdisciplinary studies, theoretical framework

## Abstract

Pedestrian route choice, the process by which individuals decide on their walking path between two locations, is a fundamental problem across disciplines. Because this behaviour is investigated from different conceptual and methodological angles, and because it strongly depends on the environmental context, it is challenging to establish a systematic framework for research. Here, by reviewing previous work, we identify four principles for pedestrian route choice that are relevant across disciplines. First, ‘information perception’ deals with how pedestrians can perceive information selectively and purposely, given the limited available information. Second, ‘information integration’ considers how pedestrians subjectively integrate environmental spatial information into mental representations. Third, ‘responding to information’ is concerned with how pedestrians tend to be attracted and repelled by specific attributes individually and how this can lead to positive or negative feedback loops across many individuals. Fourth ‘decision-making mechanisms' describe how pedestrians trade off the evidence provided by different attributes. How pedestrians perceive, integrate, respond to, and act upon information is not fixed but varies with the context. We give examples for each principle and explain how these principles shape pedestrian choice behaviours. We hope this contribution provides a systematic overview of the field and helps to spark inspiration among specialists.

## 1. Introduction

Imagine that you are exploring a city for the first time. Fortunately, you have a map that precisely shows the correct information to get you where you would like to go. However, there are several routes, including direct and short ones, and scenic ones that are indirect and pass through narrow lanes. Which route will you take? Now imagine that you visit a museum for the first time when the fire alarm starts to sound. When you are looking for a way out, you probably do not consider how scenic the way is. Instead, you may be looking for evacuation signs and where others are going, but it may be dark, or worse, rooms may be filled with smoke. When all cues suggest the same route, it is not difficult to decide in these situations. If not, you will have to somehow trade off different route properties that you are aware of. While these two scenarios are probably not an everyday occurrence, pedestrian route choice is involved in many aspects of our daily lives, such as commuting, for example. We go to work on a specific route and go home on the same or another route. This route choice has already taken place many times, it may not be a conscious decision sometimes, and it may be determined by choice inertia. Regardless of the specific mechanisms for how we select routes, they shape our spatial behaviour.

The above examples illustrate realistic route choices people may face. Route choice is the spatial choice pedestrians make between a set of alternatives with the goal of reaching the desired destination, a process people have to deal with on a daily basis or in emergencies [1,2]. It is regarded as one of the fundamental abilities of humans and is processed automatically within the brain and without the necessity of explicit thoughts in many cases [3]. The cognitive process of route choice is relevant to motor vehicle operators, cyclists, pedestrians and other transport users. Three reasons motivate us to focus on pedestrians. First, compared to other transport users who typically travel on road or other transport networks, pedestrians access a wide range of facilities, including commercial, residential, educational, and entertainment venues, for different reasons meaning their movement is more widely relevant to research areas beyond transportation, including architecture, safety engineering and retail, for example [4]. Second, compared to car drivers who cannot leave the road to take shortcuts, pedestrians are much less constrained by traffic rules and legal regulations and thus have a high degree of movement freedom and choice flexibility, which poses another big challenge for modelling. Third, walking is considered to be one of the most sustainable and green transport modes, especially in cities [5]. An understanding of pedestrian route choice can help the development of pedestrian facilities that provide attractive and efficient walking environments which are an essential requirement for sustainable urban transport and have additional benefits in terms of public health and avoiding social isolation [6]. Therefore, we focus on pedestrian route choice here.

**Figure 1.**
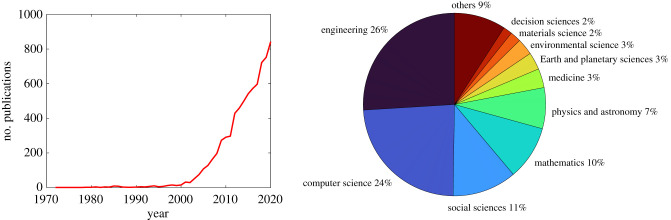
Visual illustration of analysis of research on pedestrian route choice. The line chart illustrates how the number of publications per year on this topic changes over time. The pie chart shows the frequency of the research based on the discipline. Data source: Scopus (accessed 5 November 2021).

Pedestrian route choice has long been a research topic and has drawn much attention in recent decades. As shown in figure 1*a*, the number of publications on this topic has been increasing substantially since the early 2000s and over 7000 studies have contributed to this research object. When reviewing work on pedestrian route choice, we found two main reasons that make it complex.

The first reason is that researchers from different disciplines are working on different topics related to pedestrian route choice using a variety of approaches. Figure 1*b* illustrates the percentage of all publications on this topic in different disciplines. For example, transportation scientists may develop route choice models calibrated on empirical data that involve interactions between pedestrians and vehicles with the goal of predicting future traffic conditions which in turn will support transportation control and planning in cities [7]. By contrast, neurologists might be interested in physiological structures responsible for spatial cognition, such as the parietal cortex, and may conduct controlled experiments using neurological methods [8]. Furthermore, pedestrian route choice plays a different role in the framework of each research topic. In pedestrian dynamics, pedestrian route choice is a ‘tactical-level’ decision on which route to use, distinguished from higher level ‘strategical-level’ decisions on selecting destinations and lower level ‘operational-level’ decisions on avoiding collisions with others and obstacles [9]. In transportation, route choice is the fourth step in the conventional transportation forecasting model, following trip generation, trip distribution and mode choice [10]. In psychology, the cognitive process of route choice is studied in its own right, considering what information is considered and how it is processed [11].

The second reason that adds to the complexity of studying pedestrian route choice is that it can occur on different temporal and spatial scales. For example, in evacuations pedestrians are often determined to reach a safe destination as quickly and as efficiently as possible, whereas tourists may choose scenic routes that may be less direct. In terms of spatial scales, evacuations from a smoky room [12], an entire building [2], or even a whole region that is threatened by a hurricane [13], all present situations that require individuals to choose routes, but over different distances. These different spatial and temporal scales may require different cognitive processes and especially when completing longer routes, pedestrians may update their decisions several times as they acquire new information, resulting in a sequence of route choices.

Previous reviews have covered specific aspects relevant to pedestrian route choice. For example, some studies review pedestrian decision-making but only focus on a specific scenario such as wayfinding, defined to be the process of completing short routes [14], the context of evacuations [9], or transport [15]. Other researchers review models used for reproducing realistic pedestrian behaviour and cover route choice as part of this [16,17], or they analyse the external or internal factors that affect how pedestrians make spatial decisions [18]. However, all literature reviews to date consider route choice alongside other behaviours of pedestrians, often in specific contexts, and there is no study on this topic that presents a general perspective that is relevant and useful across research disciplines.

The discussion above highlights the importance of identifying the key mechanisms in pedestrian route choice and their relevance across contexts. In this contribution, we propose that the essential principles of pedestrian route choice are information perception, information integration, information response and mechanism of decision-making. We discuss how these principles are affected by and operate in different contexts. Our aim is to establish key principles in pedestrian route choice and their relevance across disciplines rather than providing an exhaustive survey of the pedestrian route choice literature.

This review is organized as follows. Section 2 consists of five parts. The first four each introduce one principle for the pedestrian route choice process and the last part discusses how pedestrian route choice depends on the context. Section 3 summarizes all principles and discusses opportunities for future research. Section 4 presents the main conclusion of this review.

## 2. Principles of pedestrian route choice

We argue that the essence of route choice in pedestrians can be distilled into four processes which we will discuss in the following: information perception, information integration, responses to information and decision-making mechanisms.

### 2.1. Information perception

Pedestrians can perceive information selectively and purposely, given the limited available information.

Information perception is the essential required first step for pedestrians to be able to represent the environment they are in when choosing a route to their destination. Two primary processes have been distinguished. First, acquiring sensations in which people gain experiences from the stimulation of a single sense organ, and second, perceptiveness in which people identify and interpret sensory information [19]. Selective attention has been identified as the dominant feature of these processes [20].

#### 2.1.1. Selective attention

**Figure 2.**
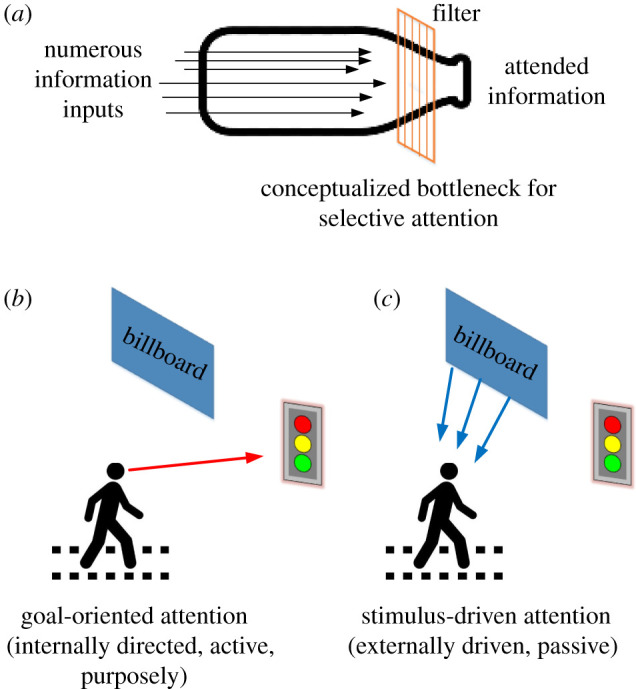
(*a*) Conceptualized bottleneck for selective attention. (*b*,*c*) Two different processes of attention.

Previous research has established that people can choose information used for the representation of environments [11]. More specially, people centre their attention, purposely focusing on details and casting irrelevant information to the side-lines of their perception. For example, buildings offer too much spatial information for pedestrians to process, meaning that they are likely to only focus on landmarks or other memorable features that are useful for their route choice task. However, whether such information is selected at an early or late stage during information processing has been discussed extensively within the field of cognitive psychology [21]. The filter model of attention, proposed by Broadbent, based on dichotic listening tests, is a typical early selection model of attention [22]. It assumes that a selective filter is needed for information processing due to the limited capacity of attention, allowing only specific information to pass through for further processing and to filter unattended information out. By contrast, late selection models of attention suggest that both attended and unattended information are processed to the same deep level of analysis until the selection occurs [23]. Regardless of the specific mechanism, pedestrians perceive information selectively to allocate limited cognitive processing resources [24], which is often described as a bottleneck (figure 2*a*).

In terms of selective attention, a key challenge is to determine which information should be attended to and which inputs should be ignored. The bottom-up (or stimulus-driven) attention and top-down attention (or goal-oriented attention) perspectives are commonly considered categories [25]. As shown in figure 2*b,c*, stimulus-driven attention is externally driven by salient features with inherent and distinct qualities that contrast with the surrounding environment. For example, pedestrians are reported to be distracted by billboards while crossing roads, even though the billboards are not relevant to their route choice task [26]. By contrast, goal-oriented attention is internally directed and allows people to allocate their attention voluntarily based on prior knowledge and current tasks [25]. In the process of wayfinding, pedestrians tend to focus on searching for signs to obtain directional clues, for example. While there are essential neurological differences between these two types of attention, they both result in the attended objects receiving preferential processing [25].

#### 2.1.2. Limited available information in pedestrian route choice

Various types of information can be available to pedestrians. Information sources can be categorized into static, which do not change with time, and dynamic, which change over time [27]. Vision is the primary sense for most pedestrians to perceive information from the environment. Human brains use binocular disparity to extract depth information from the two-dimensional retinal images via stereopsis, allowing pedestrians to estimate the distance and size of an object [28]. In this way, pedestrian can detect spatial information, such as landmarks and signs, which help their navigation and orientation. Smell is another sense pedestrians rely on to perceive information. Pedestrians can extract spatial information by comparing the input across nostrils to assess the comfort of the street environment based on pleasant or unpleasant smells or to recognize the occurrence and origin of emergencies from olfactory cues, such as the smell of smoke in fires or the acrid smell of chemical gas leaks [29]. Similarly, for hearing, interaural cues facilitate the localization of auditory signals [30]. Environmental noise is an essential factor for the perceived quality and comfort of places, and alarms and other auditory messages can alert and guide pedestrians in evacuations [31]. Differences between individuals, illness, injury or disability influence how pedestrians use their senses and thus what spatial information is available to them.

While pedestrians can perceive information selectively and purposely, the available spatial information is still limited, which can present a challenge in route choices. For example, the spread of smoke in fires or power supply failures can mean there is little or even no visibility [32]. In such circumstances, pedestrians may struggle to detect emergency signs, walls, floors, doors and stairways. Instead of visual perception, pedestrians have to depend on haptic perception to avoid surrounding obstacles and to find a route to safety [12].

### 2.2. Information integration

Pedestrians subjectively integrate environmental spatial information into mental representations.

**Figure 3.**
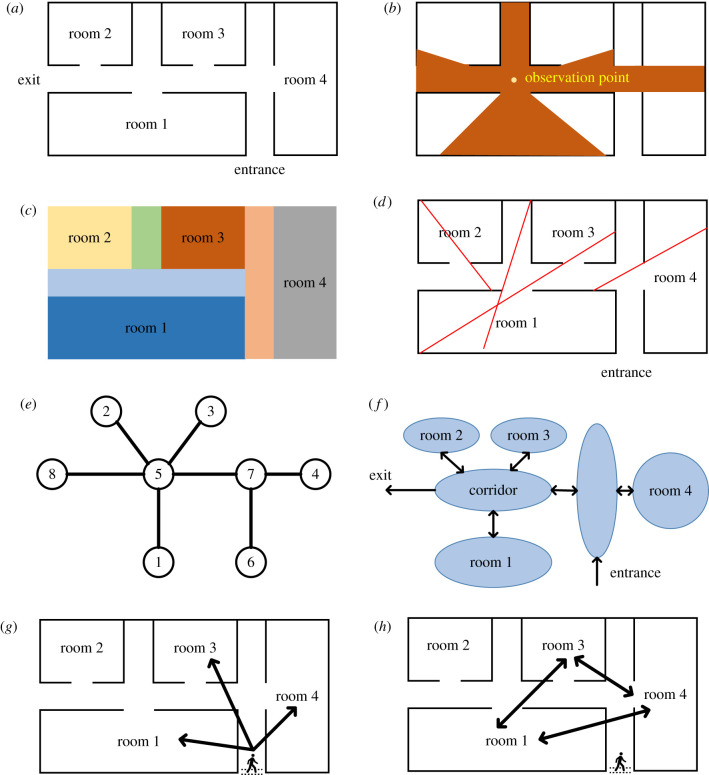
Different representations of space: (*a*) original floor plan, (*b*) isovist map, (*c*) convex map, (*d*) axial map, (*e*) network representation, and (*f*) one possible cognitive map. The remaining panels show spatial reference frames: (*g*) allocentric frame and (*h*) egocentric frame. See text for details.

After perceiving information, pedestrians need to integrate this information into mental representations of the environment surrounding them. There are many ways to represent spatial arrangements of environmental features, such as walls, rooms and signs (figure 3). Some of these representations are adopted by researchers for convenience or computational benefits, whereas others try to describe or capture the cognitive processes of pedestrians. For example, in transportation, space is often represented as a network where each node represents an intersection point, and each edge that links nodes represents the streets in cities, paths in the countryside or corridors in buildings that pedestrians can travel on [33] (figure 3*e*). Models predicting pedestrian route choice from an origin to a destination based on networks are generally developed in an algorithmic manner, such as Dijkstra's algorithm for finding the shortest route to a destination and the A-star algorithm, which determines a path to a given goal with the smallest cost [34].

Space syntax is another method for representing space, which focusses on the connectivity and integration between spatial components [35]. Three types of maps can be derived for different purposes. An Isovist map depicts the volume of space visible from any given position within the configured space (figure 3*b*), which provides a mathematical basis for analysing visual information and can be used to investigate the visual stimulus of the arrangement of interior elements for improving architecture design [36]. A convex map is the minimal set of convex spaces that covers a layout (figure 3*c*). It has been related to the social use of spaces [35]. An axial map is constructed by the least number of axial lines that cover all convex spaces of a layout (figure 3*d*), which can describe the structure of movement in a spatial setting, making it a valuable tool for studying the dynamics of social life such as the selective distribution of a population and the range of choices determining their mobility in spaces [35].

Networks and maps generated using space syntax capture objective representations of spatial environments. By contrast, the mental representation pedestrians develop of external environments is based on cognitive processes that might affect route choice. Researchers have worked toward formalizing this process by suggesting cognitive maps that capture spatial relations among features and objects, as a method of describing mental spatial representation [37]. Five typical elements of cognitive maps have been suggested: paths, nodes, districts, edges and landmarks [38]. Paths refer to the corridors, edges are limiting or enclosing features, districts are larger spaces that may be categorized according to common characters, nodes are the intersections of major paths or places, and landmarks are distinctive features that people use as reference points for their location. The concept of cognitive maps was termed by Tolman based on evidence about rats possessing clues about specific objects and their spatial relation that they obtained from the experience of previously visiting other environments [39]. Research evidence from rats also suggests that the hippocampal formation is involved in the establishment of cognitive maps and that specific cells, such as place cells and grid cells [40], play a role in spatial information integration. Similar cells that provide environmental information have also been discovered in the human brain [41].

In the process of constructing cognitive maps, two different spatial reference frames are used to structure the environmental information. One is called the egocentric (self-to-object) frame, which refers to topographical relationships between a person and the environment he/she is in; the other is called the allocentric (object-to-object) frame and it records spatial information about the location of objects relative to each other in the environment [42]. Figure 3*g*,*h* shows examples for these two kinds of reference frames. The egocentric frame is self-centred meaning the perspective depends on the current location of an individual, while the allocentric frame is founded on world-based coordinates and encodes spatial information from a stationary perspective. Both reference frames are necessary for pedestrian navigation and pedestrians can switch between them or combine them if needed [43]. Previous research has demonstrated that there is no difference between the behavioural performance of participants who are provided with either allocentric or egocentric visualizations [44], but it has been suggested that the reference-frame preference of individuals is influenced by their age [45] and gender [46]. Thus, individual characteristics are likely to influence the cognitive maps constructed for environments, but it is not clear to what extent this also influences route choice.

Since pedestrians perceive spatial information selectively and purposely, they integrate different information and construct subjective cognitive maps for episodic activities that may depend on their beliefs, experiences or attitudes, even if they have access to the same information from the same environment [47]. Therefore, cognitive maps can be inaccurate, simplified, or even distorted when compared to objective representations of physical environments (figure 2*e*,*f*) [48].

### 2.3. Response to information

Pedestrians tend to be attracted and repelled by specific attributes individually and this can lead to positive or negative feedback loops across many individuals.

Pedestrians respond to their environment based on the mental representation of it they have developed and because of their inherent response preferences to attributes of the environment. Previous research suggests attributes that characterize an environment, such as sidewalk condition, steep slopes, intersection density, distance and the number of directional changes, are relevant to pedestrian route choice [49,50]. Pedestrians tend to be attracted or repelled by specific attributes and these individual-level responses can result in positive or negative feedback loops across many individuals.

#### 2.3.1. Desirable and undesirable attributes

In general, attributes of the environment relevant to route choice can be categorized as being desirable or undesirable, reflecting the tendency or preference of choosing or avoiding a route that has a given attribute. These categories have alternatively been described as attractive and repulsive forces [51].

The exact response of pedestrians to specific attributes may depend on individual characteristics or previous experience and, importantly, the context. For example, some people prefer a less busy route to avoid others, while other people who are not familiar with a building may tend to follow the crowd because the movement of others is an important source of information. Other behaviours may be more stable across populations. One example of this is the side preference behaviour where pedestrians prefer to walk on the right-hand side or the left-hand side, to avoid conflicts in a bi-directional flow situation [52]. It has been shown that each pedestrian possesses an inherent side tendency, although this preference varies significantly with regions, suggesting it is related to cultural conventions [53]. We will discuss the importance of the context on the behavioural responses of a pedestrian to environmental attributes below.

#### 2.3.2. Positive and negative feedback

**Figure 4.**
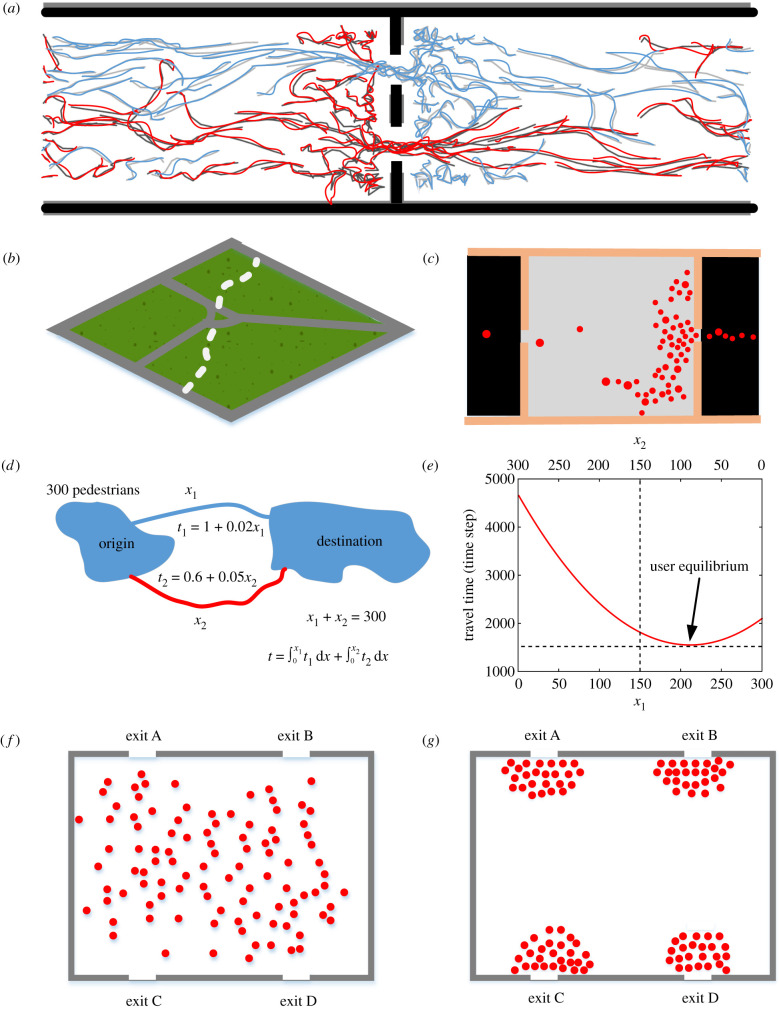
Examples of positive and negative feedback in pedestrian route choice. (*a*) Different door usage for pedestrian flows moving in opposite directions. (*b*) The spontaneous path. (*c*) Unbalanced exit usage caused by positive feedback. (*d*,*e*) User equilibrium caused by negative feedback in route choice. (*f*,*g*) Negative feedback of congestion can convert an initial inhomogeneous spatial distribution of pedestrians into a balanced distribution of pedestrians across exits during egress. (*d*,*e*) Examples given by the authors. (*a*–*c*,*f*) and (*g*) are redraw from [51,55,56] and [57], respectively.

Positive feedback is the amplification of events through recruitment or reinforcement. It is one of the paradigmatic features of collective behaviour, dynamics arising from the interaction between many individual agents [54]. A good example of pedestrian route choice is shown in figure 4*a*. Consider a passageway where two pedestrian flows are moving in opposite directions. Pedestrians can use either of two doors. While pedestrians moving in opposite directions hinder the passage through a door, it is much easier to follow people moving in the same direction through a door. Thus, over time, a slight imbalance between the doors can accumulate with increased flows in one direction gradually blocking the flow in the opposite direction through a door. This can result in pedestrians walking in opposite directions using different doors. The mechanism for this process can be active in that pedestrian choose to use the door that allows them to pass more easily, or passive in that having to avoid others walking in the opposite direction hinder progress towards one of the doors. In either case, the positive feedback could mean two doors are much more efficient than one single door that is twice as wide, in this case [55].

Another example of positive feedback is the spontaneous formation of paths by pedestrians (figure 4*b*) [56]. Some pedestrians occasionally forge a new path as a shortcut and if noticed or visible to others this new path may be attractive to subsequent pedestrians, who further reinforce the path. This positive feedback reduces the physical and cognitive costs for pedestrians [58]. While the preceding examples suggest beneficial outcomes of positive feedback loops, they can also lead to suboptimal solutions. As shown in figure 4*d*, if individual pedestrians tend to follow others while escaping a smoky room through two exits that are partially concealed by smoke, then the resulting positive feedback increases the number of the crowd moving in a specific direction and causes unbalanced and thus inefficient, or even overcrowded, exit usage [51].

By contrast, negative feedback is a process when the results of an action inhibit that action from continuing to occur. It generally promotes stability and reduces the effects of perturbations. Consider, for example, a simple network, as shown in figure 4*d*, where pedestrians have to choose either a longer route or a shorter route to reach a destination. Suppose they are more likely to choose the less busy route to lower their individual travel cost, making this preferred route busier than the other route. However, as more people choose the shorter route, it becomes congested, which incurs a time delay, making the shorter route less and less attractive compared to the longer route. As a result, the process reaches an equilibrium where there is no longer an incentive to preferentially choose either of the routes (figure 4*e*) [59]. Figure 4*f* illustrates another example where pedestrians are distributed unevenly in a room with four exits prior to the onset of egress from this room. If each pedestrian chooses an exit based on an objective estimation of the remaining travel time, then they will initially tend to choose the nearest exit. Over time, the increasing number of people clustered around exits will cause congestion and increase the estimated travel time for exits causing pedestrians to avoid exits, even if they are nearby. As a result, pedestrians will distribute approximately evenly across the available exits (figure 4*g*) [57]. This process reduces the total and individual egress travel time and thus leads to an effective evacuation.

### 2.4. Decision-making mechanism

Pedestrians perform trade-offs based on the evidence provided by different attributes.

So far, we have discussed how pedestrians perceive information, how they integrate it into mental representations, and how some aspects of environments are desirable and others are undesirable in route choice. Now, we discuss the mechanisms by which pedestrians decide on their route. There is a consensus that if pedestrians are aware of several route options, they trade off the evidence provided by different environmental attributes associated with the alternatives when making their decision. The precise process for how this trade-off is arrived at is unknown. There are two broad theoretical paradigms that are both useful for understanding and predicting pedestrian route choice. The first can be described as utility theory. It assumes pedestrians assign a value to environmental characteristics and then choose the option with the best value, potentially subject to some uncertainty [60]. The second assumes that pedestrians do not perform such optimizations but instead rely on a repertoire of simple decision strategies or rules of thumb that are known as heuristics [61].

#### 2.4.1. Utility theory

In utility models, the preference of a pedestrian for each option is assigned a quantitative value, known as a utility. The utility measures the degree to which the goals of an individual are achieved as a result of their decisions. Thus, the utility assigned to environmental features, such as exit signs, or properties of routes, such as their length, is crucial. The choice set contains all available mutually exclusive alternatives which in the context of pedestrian route choice is a finite number of options [60].

The general form of the utility function is shown in equation (2.1).

2.1 $$U_{n}=V_{n}+\varepsilon_{n}$$

and

2.2 $$V_{n}=\beta_{1}X_{n_{1}}+\beta_{2}X_{n_{2}}+\cdots +\beta_{k}X_{n_{k}}$$

For an alternative n, the utility *U_n_* consists of a deterministic component *V_n_* and a random component ɛ*_n_*. The former is calculated by combining the utilities of separate attributes associated with alternative *n*. One example for this is given in equation (2.2), where $X_{n_{k}}$ is the vector of observed attribute values and *V_n_* is expressed as a linear combination of the contribution of the *k* observed attributes, with a vector of utility parameters *β*_k_ that captures the relative weight of the corresponding attributes. The random component *ɛ_n_* can be interpreted to describe lack of information or other cognitive processes in pedestrians, or it can reflect our incomplete knowledge of the decision-making processes in pedestrians, such as not knowing all factors that influence pedestrian route choice or differences between individuals. Different assumptions about the distributions of the random utility component result in different utility models. For example, in probit models, logit models and multinomial logit (MNL) models, the random components are assumed to follow a normal, logistic and extreme value (Type I) distribution, respectively [62].

Current research into pedestrian route choice using utility theory aims to establish the relative utility of route attributes via measurements or experimentally, or it develops novel models based on the concept of utility. For example, [63] conducted an experiment on stated and revealed pedestrian exit choices and estimated the utility parameters of several attributes using four mixed logit models. Their findings suggest that the spatial distance to exits, the level of congestion around exits, and the visibility of the exit contribute significantly to the exit choices of pedestrians. In a different approach, experiments are used to test the sensitivity of pedestrian route choice to changes in different attributes. For example, [64,65] distinguish static information (time-independent), such as exit width or route length, from dynamic information (time-dependent), such as the level of congestion along different routes, and test the trade-off between these two kinds of information regarding pedestrian exit choice. An example for theoretical developments is the work by [50] who proposed a new model for pedestrian behaviour based on utility theory. In this model, pedestrians are assumed to schedule their activities, the activity areas, and the paths between the activities simultaneously to maximize the predicted utility of their efforts and walking.

#### 2.4.2. Heuristics

Despite their random component, utility models assume people hold knowledge of costs associated with all alternatives and perform an optimization across them. It has been suggested that this may not be an appropriate representation of the cognitive processes people perform [66,67] called situations where people could have near-perfect knowledge ‘small worlds’, assuming they only occur in constrained circumstances, and argued that people are more likely to use rules of thumb or heuristics to make decisions in the ‘large world’ where information tends to be unknown and cannot be measured easily.

A heuristic is a decision rule that does not seek to optimize and may ignore part of the information with the goal of effort reduction. In other words, heuristics allow people to make decisions quicker and with less cognitive effort using simple rules and inferences. It has been suggested that this reflects how individuals use cognitive shortcuts to reach intuitively correct decisions [61].

A regular criticism of heuristics is that people save time and effort with a heuristic at the cost of accuracy. However, in some cases simple heuristics are more accurate than standard statistical methods that have the same or more information. When less information or computation leads to more accurate judgements than more information or computation, these results are known as less-is-more effects [68].

Research has identified several heuristics pedestrians may use to make route choices (table 1 and see [69] for a review). One type of heuristic can be described as one-reason heuristics that assume pedestrians only use one cue (principle, rule, criteria or strategy) to compare alternatives for decision making and focus on the characteristic of the route. Examples include: (1) the least-decision-load heuristic, (2) the least-angle heuristic, (3) the shortest distance heuristic, (4) the quickest path heuristic and (5) the least costly path heuristic. Other types of heuristics focus on the relationship between the route and the environment. Examples include (1) the action continuation heuristic, (2) the initial segment heuristic and (3) the central point heuristic, (4) the hill-climbing heuristic and (5) the fine-to-coarse planning heuristic.

**Table 1.** Examples of heuristics identified in pedestrian route choice.

types	heuristics	descriptions
one-reason-heuristics	the least-decision-load heuristic	pedestrians tend to choose the route with the least number of possible decision points
	the least-angle heuristic	pedestrians tend to choose the path at an intersection which is most in line with the target direction
	the shortest distance heuristic	pedestrians tend to choose the shortest path
	the quickest path heuristic	pedestrians tend to choose the quickest path
	the least costly path heuristic	pedestrians tend to choose the least costly path
others	the action continuation heuristic	pedestrians tend to proceed with the current course of action, ignoring other alternatives
	the initial segment heuristic	pedestrians tend to choose the initial path with a later turn so that they do not have to turn for as long as possible along their route
	the central point heuristic	pedestrians tend to choose the well-known parts of a building, even if this requires detours
	the hill-climbing heuristic	pedestrians tend to complete easily obtainable subgoals that can be achieved immediately for reaching the destination
	the fine-to-coarse planning heuristic	pedestrians tend to divide the environment into different areas, undertaking rough planning when navigating between areas and fine planning within a given area

### 2.5. Context dependency

How pedestrians perceive, integrate, respond to, and decide upon information is not fixed but varies with the context.

While the principles of pedestrian route choice we have introduced above are generally valid, the detailed mechanisms relating to each principle are not fixed but vary with the context. Differences in context can be across environments, consider typical behaviours at tourist sites and railway stations busy with commuters, or over time within environments, such as the onset of a fire alarm in an office building or students gradually becoming familiar with the building on their university campus after the start of term. In the following, we discuss the contextual factors that have received the most attention in previous research: motivational state, familiarity, social influence and individual characteristics.

#### 2.5.1. Motivational state

Motivation is essential for human decision-making and motivational states are associated with different neurological mechanisms [70], which may lead to different choices when pedestrians select their route.

A good example is the route choice of tourists, commuters and shoppers. These three groups of pedestrians may consider route attributes in very different ways. Tourists for sightseeing purposes may emphasize the quality of visual attraction offered by routes that make the route entertaining or pleasant [71]. Commuters with the goal of reaching workplaces as easily as possible tend to choose the shortest possible route without inclines [72]. The route preference of shoppers varies with their motivation: hedonic shoppers like to stroll around in the shopping area while utilitarian shoppers prefer more efficient routes [73].

Another typical example is route choice in emergency evacuations where pedestrians are often under time pressure, which can give rise to a range of behavioural responses that are often described as stress. Even though stress can lead to a beneficial vigilance in information processing, higher stress levels may limit the capacity of individuals to process environmental information effectively and, therefore, ultimately lead to errors in decision making. For example, based on virtual experiments pedestrians may be more likely to select known routes and are less likely to adapt their choices, even if this leads to longer evacuation times. Bode *et al.* [65], Bode & Codling [74] and Helbing *et al.* [75] considered extreme emergencies where pedestrians are assumed to transit from normal behaviour to behavioural states where they have a stronger tendency to follow others, resulting in the unbalanced usage of exits. Haghani & Sarvi [76] compared the attributes of pedestrian route choice in normal and emergencies, finding that under normal circumstances the distance to the exit is the dominant factor affecting pedestrian choice, while pedestrians place a much higher priority on avoiding crowded exits in an emergency.

#### 2.5.2. Familiarity

Familiarity describes the spatial knowledge of pedestrians, which is acquired through experience. Human spatial memory can be distinguished into route knowledge and point and survey knowledge [77]. Route knowledge enables pedestrians to follow a sequence of connections between landmarks to reach their desired destinations without the knowledge of general interrelationships between building elements. By contrast, point and survey knowledge are related to a more general knowledge of the relative spatial positioning of elements in the environment, including awareness of their location relative to the current position of pedestrians.

Evidence from anecdotal observations and field studies reveals that pedestrians tend to follow exit routes they are familiar with and that they do not identify all available exits in fires [78] and other emergencies [79]. This preference for familiar routes can persist even when other available exits are closer [79] or others leave by a different exit [80], because pedestrians are not prepared to try an unknown route [81]. The preference of pedestrians for familiar places has been identified as an essential factor affecting pedestrian route choice [78]. One possible explanation is that people feel more comfortable in familiar spaces. The uncertainty in unfamiliar places may result in spatial anxiety, a type of anxiety about performing spatial tasks (e.g. navigation, wayfinding), which is a situation pedestrians try to avoid [82].

By contrast, when pedestrians are not familiar with an environment, they have to seek clues for their route choice. Landmarks [83], signs [84] and the movement of others [85] are specific clues, and their function in guiding pedestrians have been widely studied.

#### 2.5.3. Social influence

Social influence in pedestrian route choice describes the ways in which pedestrians change their behaviours to meet the demands of a social environment. In terms of pedestrian route choice, social influence can involve the effects of social groups and strangers on the decisions of individuals.

A social group is a number of pedestrians that are connected via social relationships, such as family ties, friendship or work relationships [86]. The members of such groups tend to stay close to each other and will thus try to walk along the same route [87]. This has implications for route choice [65]. For example, groups may have to reach a consensus on which route to choose, or individuals may follow designated or emergent leaders. Such group decisions may take longer but could also help to avoid individual errors in route choice. By contrast, strangers are pedestrians that are not connected by social ties. Previous research has established that in normal situations, pedestrians who know an environment tend to avoid busy routes and thus other pedestrians. However, pedestrians tend to treat others as a source of directional information and imitate their choices to reach a destination when they lack spatial knowledge of the environment [85]. In addition, research in social psychology suggests that strangers can develop and share a social identity (sense of unity, psychological togetherness, groupness) with each other in disasters and emergencies [88]. Social identity can motivate solidarity with strangers and enables pedestrians to identify with each other as part of a psychological crowd and then help and cooperate with each other [89]. This suggests that the influence of social groups on individual route choice can extend to strangers. However, additional research is needed to support this notion.

#### 2.5.4. Individual characteristics

In addition to contextual factors, individual characteristics are critical for determining which route pedestrians take. Previous studies have established that age and gender can affect the process of pedestrian route choice. Older adults have reduced wayfinding performance since spatial abilities (such as mental rotation and visualization) decline with age [90]. They tend to pick up environmental information with a higher level of saliency [91] and rely more heavily on egocentric reference frames compared to younger adults who use egocentric and allocentric reference frames equally [45]. Studies suggest that male participants prefer geometry cues related to the general shape of the environment and allocentric reference frames, while female participants use more landmark cues and prefer egocentric reference frames [46,92]. Furthermore, pedestrians with disabilities may perform different route choice behaviours. Pedestrians with visual impairment or blindness face both physical difficulties and increased cognitive loads while navigating and cognitively mapping new surroundings. Although adept at making up for missed visual information by improving awareness of environmental cues and navigation equipment, they may have poorly organized and integrated mental representation of their surrounding [93] and may prefer to take a longer but safer route to their destination than the shortest route [94]. Mobility-impaired pedestrians may pay more attention to accessibility instead of the distance of the route from origin to destination and tend to choose the most accessible route [95].

The contextual factors listed above illustrate the diversity of contexts and their contribution to shaping pedestrian route choice behaviours. Pedestrian route choice involves the pedestrian and the context itself as well as the interactions between them. Although the principles we identified can capture the key mechanisms and cross-domain relationships in this process, the role of these scenario factors should be considered when we apply these principles to a certain situation.

## 3. Discussion

We identify principles that capture the essence of pedestrian route choice and are relevant across disciplines, and we give examples to demonstrate how these principles shape pedestrian choice behaviour. These principles are reiterated below:

(1) Pedestrians can perceive information selectively and purposely, given limited available information.
(2) Pedestrians integrate environmental spatial information into mental representations with subjectivity.
(3) Pedestrians tend to be attracted and repelled by specific attributes individually and this can lead to positive or negative feedback loops across many individuals.
(4) Pedestrians perform trade-offs based on the evidence provided by different attributes.
(5) How pedestrians perceive, integrate, respond to and decide upon information is not fixed but varies with the context.

**Figure 5.**
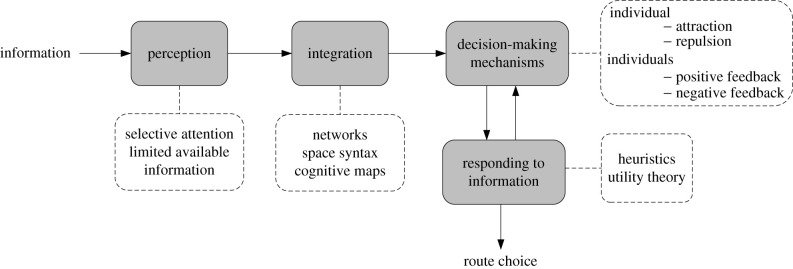
Framework for pedestrian route choice based on the principles identified here. Decision stages are shown as grey boxes. Arrows indicate the direction in which pedestrians process information, and boxes with dashed boundaries includes details on the principles we identified for each decision stage.

Figure 5 illustrates a framework for the route choice decision-making process and it relates to the principles we identified here. The route choices of individual pedestrians involve behavioural and physiological mechanisms related to information perception, information integration, responding to information and making decisions. All stages and mechanisms involved in this process can be affected by the context. The four decision stages shown in figure 5 and the processes associated with them can occur in sequence or simultaneously. For example, when pedestrians make decisions, they are still constantly perceiving new information and thus update their spatial mental representations. However, for a single route choice, pedestrians may process information according to the steps shown in figure 5 in sequence, and each stage depends on the previous stage until this route decision is completed (except for interdependent decision-making mechanisms and responding to information). Processes and mechanisms relevant to each stage can be described using the principles identified here.

These proposed principles are reflected in models that researchers have developed. For example, the knowledge-based routing model in [96] constructs a personalized cognitive map for navigation by using individual spatial memory and provided environmental information. This process can be associated with the second principle. Suppose we apply concepts of selective information perception from the first principle to this model. In that case, individuals may only perceive part of the available information for the creation of cognitive maps. In this way, our framework provides a reference point for assessing which aspects of behaviour can be added to existing or new models. Developing new models is one of the possible uses of our framework. However, we suggest its main usefulness is different and discuss this below.

Much research into pedestrian route choice focusses on specific settings and does not consider aspects that are not immediately relevant to the research question investigated. While this reductionist approach forms an important part of the knowledge generation process and may be entirely appropriate to describe behaviour in fixed settings, such as carefully controlled experiments where participants are exposed to different stimuli in the same scenario, it is important to acknowledge the limitations of this approach. For example, a utility model can have an excellent performance in pedestrian route prediction even if selective information perception or/and feedback, the first principle we discussed, is not included in the model. However, such a model may not be useful for scenarios where pedestrians can only perceive limited information. We do not suggest that the principles we identify here provide a route to establish a universal model for pedestrian route choice. Instead, we suggest that our contribution provides a framework for considering which aspects of pedestrian route choice are accounted for in a given model.

From the perspective of managing pedestrian facilities during events or emergencies, it is desirable to be able to predict or even control the route choice of pedestrians to minimize uncertainty and to maximize evacuation efficiency. Our principles provide a theoretical basis for developing route choice control or route guidance strategies. For example, taking the first principle as a starting point would suggest to selectively highlight information about particular routes. Alternatively, based on the third principle, routes could be made more attractive by changing their attributes, such as lighting or signage, or, based on the fifth principle, pedestrians could be made familiar with routes in a targeted way, as already happens on passenger airplanes. While many of these approaches are already being used or considered, we suggest that our principles provide a framework to structure and contrast strategies.

More generally, we suggest the usefulness of our principles is that they provide a frame of reference that can be used to catalogue, contrast and analyse existing and planned research on pedestrian route choice. The broad behavioural mechanisms we identify facilitate abstracting from the details of behaviours observed and contexts investigated in individual studies. As such, they provide an ideal basis for categorizing and comparing existing work and for researchers to examine to what extent their planned work is already covered in the existing literature. For example, a large body of research focuses on how pedestrians respond to specific types of information during evacuation, and if researchers categorize their work into the third principle of our framework, they can find other similar research to avoid duplication of work and find potential reasons to interpret differences in results by comparing to other work. Alternatively, as discussed above, in transportation research there are many studies that investigate the attractiveness of route characteristics in experiments or from observations, but much fewer studies investigate the second principle, subjective mental representations of environments, which possibly influences human route choice and is worth further investigation. Our principles also provide a structured theoretical starting point for interpreting or explaining observed behaviours. For example, if it is observed that pedestrians completely avoid an available route, we suggest it is useful to structure the search for an explanation by considering our principles in turn. For example, it is possible that, based on the first principle, participants selectively ignore the information provided by the researchers, or based on the fourth principle, they choose their route according to specific heuristics, disregarding other options. It may be that future research identifies principles in addition to the ones we discuss here. This would be a clear indication of how our understanding of pedestrian route choice extends.

Technological and methodological innovations will continue to facilitate research into pedestrian route choice. We suggest that three technologies in particular will shape this field of research in the coming years: virtual reality, wearable sensors and machine learning. Separately, and in combination these tools will allow examining the principles of pedestrian route choice in unprecedented breadth and detail. In virtual reality experiments participants interact with a highly controlled immersive virtual environment [97]. The fact that experimenters can control precisely what information is available to participants who may be near-stationary (e.g. on a treadmill) opens up the possibility to investigate detailed questions on what environmental features pedestrians attend to, possibly linked to neurological activity, and how different route attributes are traded off. While this experimental paradigm is already widely used and accepted in pedestrian behaviour research, a drawback is that its ecological validity should be considered carefully [98]. By contrast, increasingly available wearable sensors can continuously record the position, as well as physiological and neurological activity of pedestrians [99], making it possible to examine route choice in field studies where behaviour can be observed outside of experimental settings. Both of these approaches result in large quantities of data that has to be examined for relevant patterns. Machine learning can consume and process unstructured data and automatically determine the features that distinguish different categories of data from one another [100]. Thus, in combination with the other tools we expect machine learning will help researchers discover pedestrian decision-making patterns and determine more principles that influence human behaviour.

Pedestrian route choice is a highly interdisciplinary topic. Researchers from different disciplines are contributing to it by applying the methodologies and theoretical frameworks of their discipline. We argue our principles provide a general theoretical framework that facilitates bridging across disciplines.

## Data Availability

This article has no additional data.
